# Supplementary figures and images for: Quality considerations and major pitfalls for high throughput DNA-based newborn screening for severe combined immunodeficiency and spinal muscular atrophy
Source: PLoS One. 2024 Jun 28;19(6):e0306329. doi: 10.1371/journal.pone.0306329 (PMC11213327; doi:10.1371/journal.pone.0306329)

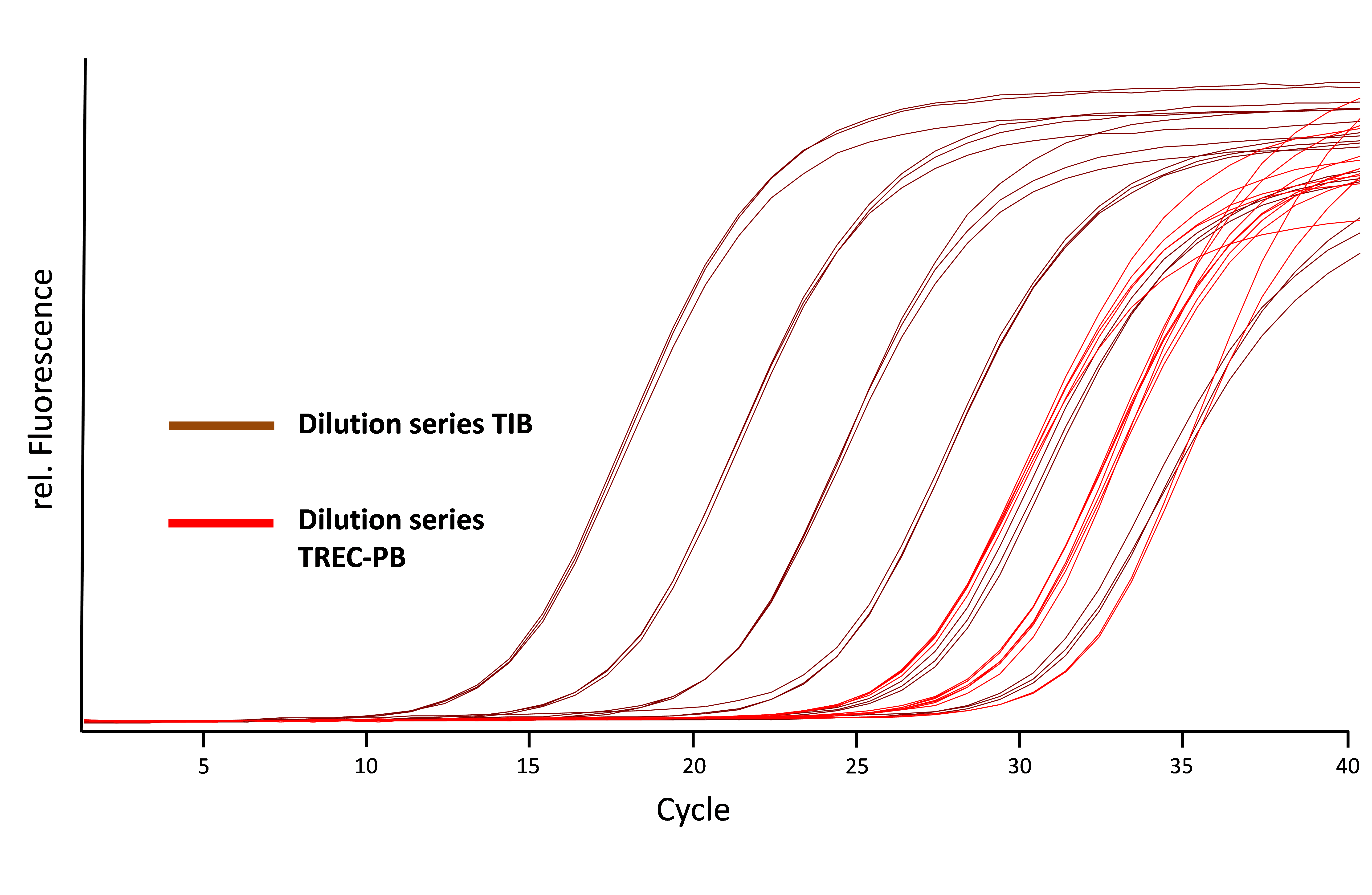

Supplement: S1 Fig — (TIF) [file pone.0306329.s007.tif]
