# Supplementary material for: Quality considerations and major pitfalls for high throughput DNA-based newborn screening for severe combined immunodeficiency and spinal muscular atrophy
Source: PLoS One. 2024 Jun 28;19(6):e0306329. doi: 10.1371/journal.pone.0306329 (PMC11213327; doi:10.1371/journal.pone.0306329)
Supplement: Attachment — Submitted filename: Rebuttal_letter_31052024.docx [file pone.0306329.s008.docx]

Dear Prof. Joshi, dear Prof. Wang and Reviewer 2,

thank you for the chance of revising our manuscript. This way we can fit it even better to Plos one and the Plos one policies. We also appreciated your suggestions that really helped us to see where we can add significant value to our manuscript and findings. We hope that we could address your points accordingly.

1. When submitting your revision, we need you to address these additional requirements.

Please ensure that your manuscript meets PLOS ONE's style requirements, including those for file naming.

- Please excuse us, for not having followed the guidelines in some points, the headings and subheadings have been corrected, the figures, figure captions, abbreviations and affiliations fixed.

2. We note that your Data Availability Statement is currently as follows: All relevant data are within the manuscript and its supporting Information files

- Thank you for clarifying what needs to be shared for a better reproducibility of the results. We have added supplements that include the data used for our calculations.

3. We note that you have included the phrase “data not shown” in your manuscript. Unfortunately, this does not meet our data sharing requirements. PLOS does not permit references to inaccessible data. We require that authors provide all relevant data within the paper.

- We initially considered this data as a minor aspect of the manuscript, however we agree that it makes sense to include all the relevant data, thus we included it as supplement.

4. Please include your full ethics statement in the ‘Methods’ section of your manuscript file.

- We agree this is an important point, that’s why we have included a section titled ‘ethics statement’ in the material and methods section.

5. Please include captions for your Supporting Information files at the end of your manuscript, and update any in-text citations to match accordingly.

- We have added the required information.

6. Please review your reference list to ensure that it is complete and correct.

- We have checked the references and are not aware of any articles retracted that we have cited.

**Reviewer #1:** This manuscript focuses on identifying the major drawbacks of current commercial systems for analyzing TREC distribution in the diagnosis of SCID and SMA. The study also proposes a rapid in-house extraction method that is comparable to commercial systems. It was found that carefully considering potential contaminating factors and defining appropriate cut-offs can minimize false results. The experiments were systematically conducted, and the data reasonably supported the conclusions.

A small drawback is that the manuscript contains many abbreviations specific to the field of study. Consequently, every abbreviation, such as PCR, needs to be defined the first time it appears.

Thus, I recommend that the manuscript be accepted, albeit with some small wording changes before publication.

- Thank you for your helpful advice, we have introduced PCR as abbreviation, carefully rechecked the manuscript and corrected abbreviation introductions. Now the introductions always appear at first use.

**Reviewer #2:** This manuscript presents a DNA based newborn screening for severe combined immunodeficiency and spinal muscular atrophy. The study holds significant promise, but several revisions are necessary to meet the quality standards of the PLOS ONE journal. Below are specific comments and suggestions for improvement.

1. Authors are advised to change the Italic writing style of Background, Methods, Results, and Conclusions and remove the colon.

- Thank you for pointing out the formatting issues, we have fixed that.

2. Combine the introduction content from lines 66-80 on page 4 into a single paragraph. Additionally, remove the numbering for titles throughout the manuscript.

- This improves the readability of the first paragraph. The combined paragraph can be found in the revised version from line 62-75.

3. Add a Data Collection subsection in the Materials and Methods section, clearly indicating the source of your data.

- We have added a section titled data collection.

4. Authors are advised to provide a detailed explanation and discussion regarding the use of freeze-thaw procedures in the Discussion section. Elaborate on how these procedures impact your results and their relevance to the study.

- We have elaborated this section of the discussion in more detail and added an additional reference between lines 413-430

5. Abbreviations need to be defined the first time they appear in the text by writing the full name and giving the abbreviation in brackets.

- We have carefully rechecked the manuscript and corrected abbreviation introductions. Now the introductions always appear at first use.

6. Bold the captions for tables and figures, and change "Figure" to "Fig." as per the formatting guidelines

- Thank you for your advice. We have carefully revised the complete manuscript based on the guidelines.

7. Avoid using bold text within the manuscript for elements such as P and M.

- We have removed the unnecessary bold elements.

8. Rename the “Perspective” section to “Conclusion” in the manuscript.

- We have renamed the section.

9. Bold all the legends in the figures and replace them with high-quality figures.

- We have reformatted the captions, the figures should be in high quality now, thanks for this valuable advice. Figures have reformatted using the PACE tool.

Thank you for considering our revisions in advance.

Best regards

Jessica Bzdok
